# Pharmacist prescriber implementation in the experiences of general practitioners, pharmacist prescribers and patients: qualitative study based on pilot trial in Slovenia

**DOI:** 10.3389/fphar.2025.1712595

**Published:** 2025-11-12

**Authors:** Matej Stuhec, Alenka Kovacic, Marjetka Korpar, Ana Banovic Koscak, Barbara Koder, Dunja Mahoric, Spela Bernik Golubic, Vesna Homar, Aleksander Stepanovic, Danica Rotar Pavlic, Eva Gorup Cedilnik

**Affiliations:** 1 Department of Pharmacology and Department of Clinical Pharmacy, Medical Faculty Maribor, University of Maribor, Maribor, Slovenia; 2 Department of Clinical Pharmacy, Ormoz’s Psychiatric Hospital, Ormoz, Slovenia; 3 Murska Sobota General Hospital, Murska Sobota, Slovenia; 4 Lekarne Ptuj, Ptuj, Slovenia; 5 Goriška Lekarna Nova Gorica, Nova Gorica, Slovenia; 6 Gorenjske Lekarne, Kranj, Slovenia; 7 Lekarna Toplek, Ptuj, Slovenia; 8 Slovene Chamber of Pharmacy, Ljubljana, Slovenia; 9 Department of Family Medicine, Faculty of Medicine, University of Ljubljana, Ljubljana, Slovenia

**Keywords:** pharmacist prescribers, qualitative study, family medicine, medication review, patients

## Abstract

**Introduction:**

Medication review services have been nationally implemented in Slovenia, and a pilot program for pharmacist prescribing has already been conducted. Qualitative evidence is required to support its integration into healthcare systems.

**Aim:**

The aim was to explore the experiences of general practitioners (GPs), patients, and clinical pharmacist prescribers regarding the national pilot trial and possible implementation of pharmacist prescribing in Slovenia.

**Methods:**

A qualitative study design using semi-structured interviews was applied. A working group developed and piloted the interview guide. Pharmacist prescribers, GPs, and patients involved in a pharmacist prescribing pilot trial in Slovenia were invited via email. Recruitment continued until data saturation was achieved. Purposive sampling was used for recruitment. Interviews were conducted between May and August 2025, recorded, and transcribed in MAXQDA. Data were analysed thematically using the Consolidated Framework for Implementation Research (CFIR). The research team agreed upon final coding. The COREQ (Consolidated Criteria for Reporting Qualitative Research) checklist was applied to ensure methodological rigour.

**Results:**

Seventeen participants were interviewed: four pharmacist prescribers, five patients, and eight GPs. Across all groups, participants expressed positive experiences with integrating pharmacist prescribers into the Slovenian healthcare system. Patients valued enhanced monitoring by clinical pharmacists and perceived improved quality of prescribing and clinical outcomes. GPs highlighted effective collaboration, particularly through medication review, as a foundation for pharmacist prescribing. Pharmacist prescribers reported professional satisfaction with monitoring and prescribing responsibilities. GPs and pharmacist prescribers expressed satisfaction with the collaborative practice agreement (CPA) developed in Slovenia and considered dependent prescribing the most appropriate model for initial implementation. Reported barriers included the absence of legislation, reimbursement mechanisms, and structured education. Both pharmacist prescribers and GPs emphasised the need for additional competencies for pharmacist prescribers in Slovenia.

**Conclusion:**

This is the first qualitative study examining a pharmacist prescribing pilot outside Anglo-Saxon countries based on experiences from a real national pilot. Findings indicate positive experiences among stakeholders and support for implementation of pharmacist prescriber in Slovenia, with implications for broader applicability.

## 1 Introduction

Pharmacist prescribing is a well-established practice predominantly in Anglo-Saxon countries, particularly in the United Kingdom (UK). In the United Kingdom, pharmacist prescribers were first integrated into the healthcare system in 2004 as dependent (supplementary) prescribers, prescribing under the supervision of a physician, and in 2006 as independent prescribers, authorized to prescribe autonomously for any condition within their clinical competence. Similar developments have occurred in New Zealand, Australia, Canada and the United States (Tonna et al., 2007; Raghunandan et al., 2021). Within the UK, pharmacist prescribing is particularly well integrated in primary care settings, including general practices (Hasan Ibrahim et al., 2022). The Collaborative Practice Agreement (CPA) is a key document that formalises the professional relationship between physicians, pharmacist prescribers, and patients within the framework of supplementary prescribing (Tonna et al., 2007; Raghunandan et al., 2021).

In contrast to the UK, where non-medical prescribing rights (prescribing by healthcare professionals other than physicians or dentists) have been widely extended to nurses and pharmacists - particularly through independent prescribing - prescribing rights in the United States, Canada, and Australia are generally more restricted and vary by jurisdiction. For example, nurse practitioners in certain regions of the US and Canada have near-autonomous prescribing authority, while pharmacists’ prescribing rights differ considerably between regions and states (Canadian Institute for Health Information, 2020; Sachdev et al., 2020; Nakhla et al., 2024; Damschroder et al., 2009).

Further qualitative and cross-sectional studies are warranted to inform the development of pharmacist prescribing within healthcare systems (Damschroder et al., 2009). In the UK, the evolution of pharmacist independent prescribing in primary care was preceded by supplementary prescribing in 2002. A study investigated the implementation of supplementary prescribing using telephone interviews with nine pharmacist prescribers, eight general practitioners (GPs), and 18 patients across six Health Board areas in Scotland (Stewart et al., 2009). Participants were generally supportive of supplementary prescribing; however, several barriers were identified. Patients reported no concerns but expressed uncertainty about what to expect during their first consultation, which initially led to apprehension. Pharmacist prescribers and GPs raised concerns regarding sustainable funding, limited professional support networks, and insufficient continuing professional development opportunities. Pharmacist prescribers strongly favoured progressing to independent prescribing, whereas GPs were more cautious, citing inadequate pharmacist prescribers’ clinical skills (Stewart et al., 2009). These findings highlight the need for pharmacists to demonstrate clinical competence to gain broader professional acceptance and underscore the importance of integrating robust clinical training within pharmacy curricula.

A subsequent study surveyed 203 general practices across Northern Ireland, focusing on clinical pharmacists qualified as independent prescribers—a standard of care since 2006 in the UK (Hasan Ibrahim et al., 2022). GPs expressed positive attitudes towards clinical pharmacists in prescribing roles. Approximately two-thirds of GPs (62.4%) reported that clinical pharmacists were qualified as independent prescribers, with 76.2% actively prescribing for patients. Most GPs indicated that pharmacist prescribers consistently possessed the clinical skills (83.6%) and knowledge (87.0%) required to deliver safe and effective care (Hasan Ibrahim et al., 2022). Respondents reported that collaboration with pharmacist prescribers improved clinical outcomes and strengthened interprofessional partnerships, increasing GP confidence in team-based care.

More recently, a 2024 qualitative study conducted remote semi-structured interviews with 20 independent pharmacist prescribers working in primary care and mental health settings (Alsaeed et al., 2025). The findings revealed that pharmacist prescribers frequently experienced low confidence in prescribing practice, particularly in mental health management. Both primary care and mental health pharmacists cited inadequate undergraduate training and limited postgraduate support as key challenges (Alsaeed et al., 2025).

A systematic review of 65 studies further explored stakeholder views and experiences of pharmacist prescribing. The majority of studies originated from the UK (n = 34), followed by Australia (n = 13), Canada (n = 6), and the USA (n = 5) (Jebara et al., 2018). Twenty-seven studies examined pharmacists’ perspectives, with fewer addressing those of patients (n = 12), physicians (n = 6), the general public (n = 4), nurses (n = 1), policymakers (n = 1), and multiple stakeholder groups (n = 14). Across contexts, stakeholders reported predominantly positive attitudes towards pharmacist prescribing, regardless of the stage of implementation. Key benefits included improved access to healthcare services, enhanced patient outcomes, optimised use of pharmacists’ expertise, increased pharmacist job satisfaction, and reduced physician workload. Reported barriers included insufficient organisational support, limited diagnostic skills among pharmacists, restricted access to patient records, and funding constraints (Jebara et al., 2018). These findings suggest that strengthening clinical competence and addressing organisational barriers are central to successful implementation.

Outside Anglo-Saxon countries, pharmacist prescribing remains very limited. In Slovenia, clinical pharmacy services are expanding in ambulatory and primary care settings and are reimbursed nationally (Stuhec, 2025a). In Slovenia, medication reviews are conducted exclusively by clinical pharmacists who have completed a 3-year specialization program. Since 2016, this service has been nationally reimbursed and implemented in nearly all primary care settings, thereby ensuring access to clinical pharmacist specialists. GPs can refer patients to clinical pharmacists using a standardized referral form. Clinical pharmacists are integrated within primary care teams, which provides a strong foundation for the implementation of advanced clinical pharmacy services, including pharmacist prescribing (Stuhec, 2025a; Stuhec and Zorjan, 2022). Clinical pharmacists typically review medications and monitor patients, but lack prescribing rights. Evidence suggests that these services reduce medication-related problems and improve patients’ quality of life (Stuhec, 2025a; Stuhec and Zorjan, 2022). These developments provide a favourable foundation for implementing pharmacist prescribing in Slovenia, including the introduction of supplementary (dependent) prescribing, which is currently in progress (Stuhec, 2025b).

To date, no qualitative study based on experiences from national pilot trial has explored the perspectives of key stakeholders—patients, GPs, and pharmacist prescribers—on pharmacist prescribing outside the Anglo-Saxon context. Such research is essential to inform the broader implementation of pharmacist prescribing across Europe and beyond. The present study, therefore, aimed to investigate stakeholder perspectives on the introduction of pharmacist prescribing in Slovenia, employing a semi-structured qualitative study design.

## 2 Materials and methods

### 2.1 Participants

This study included participants from the Slovenian national pilot trial of pharmacist prescribers. The pilot trial was conducted across four primary care settings in 2024–2025 and involved 23 GPs, four pharmacist prescribers, and 119 patients. Pharmacist prescribers had already provided type 3 medication reviews in these settings for several years, establishing collaboration between pharmacist prescribers and GPs. Medication review type 3 by clinical pharmacists has been reimbursed in Slovenia since 2016 (Stuhec, 2025a).

Within the pilot trial, pharmacist prescribers were authorised to prescribe medicines in collaboration with GPs, based on the CPA document and disease-specific protocols (Stuhec, 2025b). They provided medication reviews, issued prescriptions, and monitored patients for 6 months, until the conclusion of the trial (Stuhec, 2025b). The aim of the pilot was to evaluate the clinical and humanistic outcomes of this collaboration and to assess its potential for wider implementation in the Slovenian healthcare system (Stuhec, 2025b). The quantitative results of the pilot trial were positive, including quality of life and clinical outcomes and are reported in a separate publication (Stuhec et al., 2025). Pharmacist prescribers prescribed medications through the CPA protocol for 10 specified conditions, as defined in the protocol. Prescribing was authorized only after diagnoses were confirmed by GPs, and all prescriptions required digital signatures from the GPs before dispensing (no emergency or acute prescriptions were allowed). Patients and GPs could withdraw from the collaboration at any time during the study. Pharmacist prescribers adhered strictly to the clinical protocols established prior to the pilot trial. Detailed descriptions of the clinical protocols and the CPA protocol are provided in our quantitative paper (Stuhec et al., 2025).

For the present study, a dedicated working group of five clinical pharmacists with clinical and research expertise and four GPs was established. All members brought both research experience and clinical expertise, including backgrounds as professors, practising clinical pharmacists, and GPs. This timeframe was chosen because the pilot project was conducted from November 2024 to June 2025. Qualitative interviews for this study were conducted from May 2025 to August 2025, and the GPs, pharmacists, and patients who were interviewed all participated in the pilot study. Participants were purposively selected among those involved in the Slovenian national pilot trial.

### 2.2 Study design

A qualitative study design using semi-structured interviews was employed. Recruitment continued until data saturation was achieved, using purposive sampling. Following the guidance of Francis et al., we used the data saturation method to justify the sample size (Francis et al., 2012). The researchers included four pharmacist prescribers, five patients, and eight GPs (17 participants). Four to five participants were recruited from the four primary care settings. Two primary care settings were located in the eastern regions of Slovenia, and two were in the western areas.

The working group developed and piloted the interview guide. The study used the updated 2022 Consolidated Framework for Implementation Research (CFIR) to guide data collection and thematic analysis across five domains: innovation characteristics, outer setting, inner setting, individual characteristics, and implementation process. The framework provided a structured approach to identify facilitators and barriers to clinical pharmacist prescribing implementation within Slovenian primary care settings (Damschroder et al., 2022). The COREQ (Consolidated Criteria for Reporting Qualitative Research) checklist was used to ensure methodological rigour and an appropriate study design (Skolarus et al., 2017). In 2024, the Slovenian National Medical Ethics Committee granted ethical approval (16 October 2024; N#0120-330/2024-2711-3).

### 2.3 Recruitment

Pharmacist prescribers, GPs, and patients involved in a pharmacist prescribing pilot trial in Slovenia were invited to participate via email and telephone. All participants provided signed consent to take part in the study. Based on their activity in the pilot trial, the researchers decided to include all four pharmacist prescribers and eight GPs. GPs from each primary care setting were invited: those who had referred the highest number of patients to the pharmacist prescriber and those with the second lowest number of referrals. This strategy was designed to balance perspectives and capture reasons for high and low referral rates to the pharmacist prescriber. The pharmacist prescribers selected patients individually. Only patients without significant health impairments that could affect interview participation were invited.

### 2.4 Interviews and data collection

The semi-structured interviews were conducted between May and August 2025, recorded, and transcribed in MAXQDA®. The working group developed the interview questions based on CFIR constructs, previous papers and experiences from the Slovenia pilot trial. Semi-structured questions were designed to minimise reporting bias. The working group prepared a set of common and group-specific questions. General questions focused on demographic data, including age, experience, setting, location, and gender. Specific questions addressed experiences, satisfaction with the service, implementation, outcomes, education, and reimbursement. The draft proposal was piloted among members of the research group. It was then discussed at working group meetings, where the final interview questions were confirmed. All working group members voted to approve the final version in February 2025. The complete list of questions is provided in Supplementary Material S1.

All interviews were conducted by two researchers who had not participated in the pilot trial (one GP, Eva Gorup, and one clinical pharmacist, Dunja Mahoric) under the supervision of the other working group members. Both researchers did not participate in the pilot trial as a pharmacist prescriber or GP. Both researchers have an interest and experience in qualitative research in primary care. Interviews were conducted by telephone or Zoom, audio-recorded, and transcribed into MAXQDA®. Data were collected online (at participants’ homes) or in primary care centres (patients). Each interview began with an explanation of the study objectives, followed by collecting participants’ background information, including their care setting and professional roles and responsibilities. Subsequently, questions were posed regarding pharmacist prescribing and its implementation. Interviews concluded with a discussion of perceived barriers to implementation.

Both researchers conducted one pilot interview with a GP (a working group member) before conducting subsequent participant interviews. Each interview lasted between 20 and 60 min. To minimise reporting bias, a second researcher was present at each interview to take comprehensive notes and manage the recording. No prior relationship was established between the researchers and the participants before the study commenced. GPs and pharmacist prescribers knew that Eva Gorup and Dunja Mahoric worked within the Slovenian healthcare system. Still, they had not collaborated with them during the pilot trial in primary care settings.

No follow-up interviews were undertaken.

### 2.5 Data analysis

The data were analyzed using a thematic qualitative content analysis, applying a framework approach guided by the CFIR. A hybrid inductive–deductive coding approach was employed. Inductive coding allowed themes to emerge directly from the data, ensuring that participants’ experiences and perspectives shaped the analysis, while deductive coding was guided by the CFIR constructs to align findings with established implementation domains. Interviews were imported into MAXQDA® and transcribed using the built-in transcription function. Participants were assigned numerical identifiers. Codes and themes were generated from the transcript data within MAXQDA® and manual checking.

Two researchers (Eva Gorup and Dunja Mahoric) independently coded the transcripts. The analysis followed a systematic, iterative process beginning with data familiarisation, generation of initial codes, identification of preliminary themes, refinement of these themes, and finally defining and naming the themes to inform the study report. The researchers derived themes from the data. In cases of disagreement, the researcher, MS, served as the final decision-maker. Discrepancies were solved based on the negotiated census (calculation was not used). The working group met to ensure consistency between the data and the findings, and the research team reached consensus on the final coding framework. A coding tree was applied throughout the analysis.

Participants did not review the transcripts or codes. Two researchers double-coded to ensure trustworthiness. Demographic data are presented in tabular form, while codes and identified barriers are reported in tables and narrative text.

## 3 Results

Seventeen participants were interviewed: four pharmacist prescribers, eight GPs, and five patients (Table 1). The table presents participant characteristics. Interviews lasted an average of 30 min.

**TABLE 1:** Participants characteristics.

Clinical pharmacists (n = 4, PH1-PH4)		
Gender	Female Male	3 1
Location	Eastern Slovenia Western Slovenia	2 2
Years of experience as clinical pharmacist working in primary care clinics	≤10 years 11–20 years ≥20 years	0 4 0
General practitioners (n = 8, GP1-GP8)		
Gender	Female Male	6 2
Location	Eastern Slovenia Western Slovenia	4 4
Years of experience as a general practitioner in primary care clinics	≤10 years 11–20 years ≥20 years	2 4 2
Patients (n = 5, PT1-PT5)		
Gender	Female Male	1 4
Location of primary care setting	Eastern Slovenia Western Slovenia	2 3
Patient age	Between 58 and 83 years	

We mapped the emerging codes according to CFIR domains (Supplementary Material S2—codebook). The COREQ checklist is included in Supplementary Material S3-COREQ. Simplified Flow Diagram of Data Collection and Analysis is included in Supplementary Material S4.

### 3.1 Innovation

#### 3.1.1 Innovation source

Pharmacist prescribers were familiar with international models of pharmacist prescribing as expected.

PH2: “… I am familiar with dependent prescribing abroad and independent prescribing, mainly in the UK… and New Zealand.”

#### 3.1.2 Evidence-based

Pharmacist prescribers described following the pre-prepared protocols when prescribing for specific clinical conditions and diseases. They stressed that both the pharmacist prescribers and GPs follow the same guidelines in prescribing:

PH1: “Both GPs and pharmacist prescribers use the same guidelines. We read the same guidelines. So here I don’t think there is a difference.”

#### 3.1.3 Adaptability/acceptability

All interviewed stakeholders viewed pharmacist prescribing as positive. Pharmacist prescribers noted that, with the system of clinical pharmacists already working with primary healthcare clinics and doing medication reviews, the basics necessary for establishing pharmacist prescribers were already present.

PH1: “Slovenia is a country that already has clinical pharmacists in primary care settings … [so] this system is already in place.”

GPs repeatedly stated they found the intervention acceptable and were even enthusiastic about it.

GP4: “I believe this is the future.”

At the same time, they believed not every GP would feel the same:

GP1: “We younger [GPs] find it somewhat easier to accept this collaboration than our older colleagues, or maybe the more experienced people don’t need as much help.”

*GP3: “There will have to be a big mindset shift, necessary for all of us. But that could be positive for us and the patients*.”

They described how their perspective shifted during the pilot:

GP1: “I think once things started to develop, we all realised this is something positive.”

They stressed that GPs’ participation had to be voluntary and up to the level at which they felt comfortable.

GP4: “I support it, but it has to be consensual, in the sense that the GP has to agree to it.”

*GP5: “I think it’s good for the GP to have a sort of an overview of it, not just completely letting it go over to the pharmacist prescriber.*”

The patients also voiced general approval.

PT5: “I liked it a lot. It’s an advantage.”

One patient described that they found the pharmacist’s intervention more acceptable because the GP referred them to the pharmacist prescriber:

PT3: “I think it’s good that the GP refers you to the pharmacist prescriber. [You have] more trust … because your GP knows this person.”

#### 3.1.4 Complexity

Participants described various issues that made the intervention more challenging to implement. The pharmacist prescribers encountered logistical difficulties that made the prescribing process more complex.

PH3: “My biggest barrier is that I’m not at the primary care settings all the time … if nothing else, you can discuss things over coffee breaks … Right now, everything is discussed in meetings … which means additional burden for me.”

GPs didn’t view the innovation as complex. Some felt that it did require some more time from them:

GP7: “All the same, it did take a little more of my time.”

#### 3.1.5 Relative advantage

Both patients and pharmacist prescribers believed that the innovation was advantageous:

PT5: “This is faster. So, you don’t need the GP.”

The pharmacist prescribers stressed that the most significant shift was in offering continuous pharmaceutical care:

PH3: “The biggest benefit of this project is moving from one-time pharmaceutical advisory to … actually continuous pharmaceutical management, which is a big step forward. It is priceless … to see the patient's response, how this actually looks.”

The GPs also noticed the advantages of the continuous care:

GP7: “She [the pharmacist prescriber] monitored those patients more regularly than I in my clinic … we usually hand over this responsibility to the patient. Call when you run out of meds. Call … so we can do lab control. But now all this was done by the pharmacist prescriber, which was great.”

*GP6: “I think this might help with patients telling some more about any supplements they’re taking or anything … if they go and see someone several times, not just once*.”

### 3.2 Outer setting

#### 3.2.1 Legislation

Pharmacist prescribers were keenly aware that, according to current legislation in Slovenia, they did not have the prescribing authority.

PH4: “Currently, the legislation does not allow prescribing for pharmacists … A change of legislation will be needed … as well as the division of responsibility between a pharmacist prescriber and a GP.”

GPs mentioned the need for a report from the pharmacist prescriber they collaborated with. They also stressed that the legislation should permit the pharmacist prescribers to be able to access patient data:

GP3: “I believe it’s very important that they have access to all patients’ medical data, because that’s the only way it’s going to be safe.”

Patients agreed with that:

PT3: “I think it’s important that the pharmacist prescribers have access to my data.”

#### 3.2.2 Professional responsibility

Pharmacist prescribers accepted that prescribing would mean more responsibility for them.

“If we implement this system in Slovenia, the responsibility for the pharmacist is certainly going to be bigger.”

Some GPs felt that if a pharmacist prescribed a drug, pharmacist prescribers would have to carry the full responsibility.

GP1: “I think everyone is responsible for their own prescribing.”

However, some were not so sure, mainly because of the difference between the dependent and independent prescribing:

*GP6: “*In *a way, everyone should be responsible for prescribing. But still, the pharmacist prescriber is part of our team; they are not GPs, so I don’t know how that would work.*” And: *“For now, we still have the complete picture and responsibility and the last word on whether to send the prescription out.”*

The patients were less interested in the division of responsibility between GPs and pharmacist prescribers:

PT3: “I don’t care if it’s Ms. X [pharmacist prescribers] who prescribes, or the GP, the main thing is that it works.”

#### 3.2.3 Patient acceptance and expectations

Pharmacist prescribers felt that patients needed an explanation about the new pharmacist role:

PH3: “Sometimes … some people had doubts, they wondered whether they’d still be able to go to their GP. We must let everyone know that we don’t interfere with GP-patient relationships, but it’s just the pharmacist’s support.”

*PH1: “I think it’s important [the patients] are informed enough about the pharmacist prescriber role*.”

They believed there was additional benefit for the patient, however:

*PH3: “GPs are also busy. … They don’t manage to explain some [drug-related] things so clearly to [patients]. So I think it was some additional empowerment for patients.*”

However, all stakeholders believed the patients accepted the new service well.

GP 1: “The patients were pleased with one more expert focused on medications.”

GP2: “Patients saw this as an added value.”

GP5: “Patients were thrilled because she really focused/took her time with them.”

Pt3: “I liked it a lot.”

#### 3.2.4 Quality of care

Pharmacist prescribers believed the ability to prescribe would improve access to healthcare for patients:

PH1: “With this, we improve access to healthcare.”

GPs believed pharmacists’ prescribing helped process cases more quickly:

GP6: “I think everything went quicker. Before, I asked for a review, which took me a few days to deal with. However, we spoke on the phone and immediately dealt with it.”

However, they also believed that having pharmacist prescribers on the team improved the quality of prescriptions.

GP4: “It turned out to be a powerful tool in good care for our patients.”

GP6: “I think two heads are better than one. Everybody has their point of view, everyone knows the patient from another angle.”

There were other ways in which, according to this, prescribing improved the quality of care:

GP1: “[The patients] had some time from my referral … to think about additional questions [for the pharmacist prescriber].”

GP5: “If we both, the GP and the pharmacist prescribers, say the same, the patient finds it easier to accept, they may trust some change more.”

One GP did add a caveat:

GP8: “I see a bigger impact on quality of care than on the quantity of work done.”

### 3.3 Inner setting

#### 3.3.1 Collaboration between pharmacist and GPs

The pharmacist prescribers and GPs stressed that they have collaborated and had a good experience.

PH2: “This collaboration is based on mutual trust from before.”

GP2: “We already collaborated well before in a medication review form, if I needed advice for any patient about any medications.”

All pharmacist prescribers also mentioned they felt accepted by the GPs and were an equal part of the team.

PH4: “All the GPs who collaborated with me were interested … motivated to collaborate.”

PH2: “I always felt well and that they accepted me as an equal.”

One of the GPs stressed that the pharmacist prescribers and she worked as a team, mutually encouraging each other towards better results:

*GP7: “We* encouraged *each other, which was also good. She [said] I think we should try to go on here [*referring to increasing medication dose*], and I said yes, I think so too, and we agree. Great, let’s go on.”*

GPs had different opinions on which patients should be referred to pharmacist prescribers for prescribing. Patients with newly discovered chronic diseases would need a GP visit first:

*GP7: “With those you just discovered, you usually have to do some other things alongside. You may have to give them a referral to something. You have to do a clinical examination. … So maybe this would be less useful than with someone who has had [a disease] for a while and you realise it’s not optimally managed.*”

One GP preferred to refer simpler patient cases:

GP8: “These were the cases where I would probably do it quicker. Though it was nice to have them done by someone else, it was nice to have them do the follow-up, so increasing and decreasing [the dosage]. … I didn’t refer any complicated cases, but specifically some cases I wanted to have optimized.”

While others would preferentially refer more complicated cases:

GP1: “I think it’s like referring to secondary care. You could refer everyone who has stomach troubles to a gastroenterologist, or you can solve things in your own clinic and not crowd other clinics.”

Finally, several GPs pointed out that pharmacist prescribing played a different role in different patients:

GP3: “These are two different areas. One is prescribing and one is advising.”

The GPs were not convinced that, at the moment, as things were set up, the pharmacist prescribing was time-saving for them:

GP8: “Really, there is no time saving, because you have to think about it, write the referral, and you still have to keep an eye on it.”

GP1: “Just signing [and not having to write] the electronic prescription doesn’t present a relief. But if someone did it from beginning to end, that would be a relief.”

Both GPs and pharmacist prescribers were confident that collaboration was necessary; however:

GP3: “I think [collaboration] is essential for them and us. [The pharmacist prescriber] and I often spoke about that, that she only now, when she collaborates with us, sees through our eyes.”

PH1: “I completely understand that in the beginning, when there is a new task and competencies … that some GPs are more reserved about it … But I think the opposite, this strengthens the GP’s role.”

The patient also felt GPs and pharmacist prescribers collaborated well, hinting at a sense of safety and more streamlined care:

PT5: “I believe they collaborated well.”

PT3: “She consulted my GP and told me what is best for me. So I didn’t need to go see my GP as well.”

#### 3.3.2 Communication

Communication between stakeholders was an essential part of the pilot project, and took both time and effort.

PH3: “In the beginning, we had a lot of direct contact, to arrange who would call the patients and things like that … ”

GPs felt communication - knowing what was prescribed and why - was essential to be comfortable with pharmacist prescribing.

GP1: “I have no problem with it [pharmacist prescribing] as long as I get some report … so I can see why they did it.”

GPs also appreciated direct, immediate communication with the pharmacist prescribers:

GP6: “We had no problem… The pharmacist prescribers have a constantly available cell phone, and you can get them immediately.”

*GP3: “She [the pharmacist prescriber] always called that she prescribed something … She always let me know and explained why she decided that way.*”

A GP noted that sometimes communication was difficult because of physical distance:

*GP4: “There are some communication barriers, because we are not together in the office.*”

The pharmacist prescribers did feel that communication from the GPs’ side was not sufficient:

PH4: “I couldn’t easily follow, when someone did get the prescription and when they started taking medications … the communication was inadequate.”

Both GPs and pharmacist prescribers believed that communication should be recorded and traced in electronic medical records. This would make it easier to follow patients and coordinate care.

GP4: “A problem of this communication in a wider sense is that this information should be saved in the long run … We need long-term follow-up and a suitable note in the chart.”

PH3: “We need an implementation of this into electronic records … so that when you open up the patient, you can see that they are collaborating with a pharmacist prescriber.”

The patients were delighted with pharmacists taking the time to communicate with them:

PT3: “I like that she calls every so often, and we can talk.”

PT1: “She also called me once a month to see how things went.”

The pharmacist prescribers felt they also needed to communicate and share experiences to help each other in complex cases.

PH3: “I think we share experiences. That’s very welcome … Sometimes it means a lot to consult someone.”

#### 3.3.3 Available resources (technical and administrative support)

The pharmacist prescribers stated they would require better support, not only administratively but also technically, to make appointments with patients.

PH2: “Technical support … could improve communication between GPs and pharmacist prescribers, and support making appointments, maybe some things could be done automatically.” And: “The pharmacist prescriber should have a chance to keep a chart that the GP could also see.”

PH3: “I missed some nationwide software or application. We still create reports outside these applications … we must retype a lot.”

GPs agreed:

GP4: “If the reports were in the national electronic record, everyone who met this patient would be able to open it and see it.”

#### 3.3.4 Process structure

Each team adjusted the pharmacist prescribing process to their existing working collaboration. GPs interwove the referrals to pharmacist prescribers into their daily work and worked out pathways to refer patients in their clinics and a way to review the pharmacist’s recommendations.

#### 3.3.5 Effectiveness

Both GPs and pharmacist prescribers considered the effectiveness of pharmacist management in the pilot study. Both groups were realistic, stressing that it was not to be expected that management could be optimized for every patient.

PH4: “It’s impossible to optimize everything how we want it.”

PH3:” I think there were a few more complex cases. Maybe it didn’t go the way we wanted. We pharmacist prescribers must get used to the fact that sometimes patients aren’t the way we want.”

Some GPs and pharmacist prescribers believed that the intervention might improve patient adherence. GPs also felt it was beneficial because another health professional gave the patients the same information they did, giving them more confidence.

GP5: “For example, for neuropathic pain, I found it really good because I think they [the patients] need to hear information about it more than once to really trust the drug, and so that went really well and I was happy.”

However, one GP commented that they would have liked for the pharmacist prescribers to manage more patients if they wanted to relieve the GP clinics in any way:

GP8: “I don’t see a huge advantage, because the quantity is too small … We have to prescribe enormous amounts of meds in a working day, and here the pharmacist prescribing doesn’t even show up … It would be different if [the pharmacist prescribers] were there just for my patients.”

The patients also found pharmacist prescriber interventions effective:

PT4: “[It was good.] Before, I kept swallowing those drugs, without results.”

### 3.4 Characteristics of individuals

#### 3.4.1 Motivation (attitudes towards prescribing/responsibility)

Pharmacist prescribers were keenly aware of the increased responsibility that would come with additional competencies.

PH1: “If this system gets implemented in Slovenia, the pharmacist’s responsibility will naturally be bigger.”

PH3: “In some cases, this [confirming prescriptions by the GP] is good, because we still have some other opinion backing us, we feel safer.”

However, practice enabled them to feel more confident:

PH4: “You carry more responsibility, but the longer the pilot went, the less I worried about it.”

#### 3.4.2 Trust in the pharmacist

Both GPs and patients had gained trust in the pharmacists’ skills.

PT1: “I trust her one hundred per cent.”

One patient did add that new symptoms would prompt a consultation with the GP rather than the pharmacist prescriber:

PT3: Only if I had something new, some complication, something different, then I would first talk it over with the GP … ”

The GPs were confident that pharmacist prescribers could adequately prescribe therapies for conditions where the effect of treatment was easily measurable:

GP1: “I believe pharmacist prescribers could manage therapies for conditions requiring regulation of clinical parameters, such as hypertension, statins, diabetes, gout, and possibly some psychiatric treatments.”

One GP stressed that they felt the pharmacist prescriber was paying more attention to adjusting treatment to individual patients than sometimes other GPs did:

GP2: “At present, I sometimes trust pharmacist prescribers more than certain specialists, because prescriptions are often given indiscriminately when patients enter or leave a hospital. With pharmacist prescribers, the approach seems much more individualized.”

#### 3.4.3 Knowledge and expertise

On the whole, patients believed the pharmacist prescribers were experts in medications and felt safe with their expertise:

PT2: “The pharmacist prescriber can explain side effects better than the physician.”

The GPs believed pharmacists to be both experienced and knowledgeable in their field, and were keen to exchange experiences with them.

GP6: “Our pharmacist prescribers have a lot of experience, covering all the GPs in our area … ”

GP2: “I usually accepted most [of the pharmacist’s recommendations], because she has more pharmacological knowledge … I learned a lot from her.”

They did underscore some limitations, since pharmacist prescribers might not confidently diagnose new conditions, and might not be able to perform a differential diagnosis of some symptoms:

GP3: “I only maybe have some reservations about titrating medications for asthma or COPD … Sometimes people can have dyspnea, and it’s basically an infection … this needs some more diagnostics, not just titration of therapy.”

Pharmacist prescribers themselves were well aware that they needed extensive knowledge, additional education, and a lot of practice. However, they were not equally confident in all areas.

PH2: “Patients are very different … It depends on which group of patients you prescribe for, and which area you are more expert in. It’s not the same for everything.”

And

PD4: “Practice is most important … the more cases you do, the easier it gets.”

But they also enjoyed the challenges of learning new knowledge and skills.

PD2: “Now I understand their workload [GPs]. I see what it means to persuade a patient. And also, when writing reports, you really [should] focus on the key aspects of therapy—those urgent changes or elements that truly stand out.”

#### 3.4.4 Interprofessional relationships

Both GPs and pharmacist prescribers established the collaboration based on a good interpersonal relationship that had existed since before the project. GPs, in particular, stressed that they could hand over some of the prescriptions because they trusted the pharmacists they worked with.

GP4: “It seems there has to be a sort of individual trust. I completely trust our colleague [pharmacist prescriber]… she is a top expert, she goes beyond … This trust has to be built.”

GP5: “I have excellent experience with our [pharmacist prescriber]… has a lot of knowledge … But I can’t say what others are like.”

PH4: “I really feel accepted in this healthcare centre … we built trust between ourselves and are equal partners.”

### 3.5 Process

#### 3.5.1 Engagement

Patients at first engaged in the pilot on the instigation of their GP, but were happy to participate further after they realised what it was about:

PT4: “At first, I didn’t know what this was about, who it was meant for. But then … I had a feeling people knew what they were doing.”

#### 3.5.2 Monitoring and evaluation

Pharmacist prescribers appreciated the chance to follow up with the patients and monitor the effectiveness of their prescriptions. They found it rewarding and believed that it upgraded the quality of management, though they also felt the length of the pilot study was too short to achieve marked improvements in some of the quality indices.

PD4: “*We had a chance to do a follow-up, and so we were a lot more involved in optimisation of the therapy, which is certainly an upgrade. In fact, I think this is an essential step forward*.”

GPs and pharmacist prescribers felt that objective quality indices should be monitored to ensure safe prescribing.

#### 3.5.3 Sustainability

Though they supported the innovation and believed it was safe and useful, GPs were worried about the long-term sustainability of this approach, mainly because they worried there were too many patients for the current process of pharmacist prescribing:

GP2: “*I don’t know if this is sustainable in the long-term. I don’t think so*.”

They believed access to pharmacist prescribers would have to be limited in some way:

GP1: “*If we just open the door … for everything, this will be a lot of work for the pharmacist prescribers*.”

Pharmacist prescribers stressed the need for technical and administrative support if they wanted to expand the scope of their work. Still, both agreed that many more pharmacist prescribers would be needed to ensure sustainable implementation.

GP8: “*The problem is because we have so much of this [prescribing] to do … If we wanted to go on with this, yes, we would need a great deal more of these pharmacist prescribers.*”

#### 3.5.4 National implementation

Both pharmacist prescribers and GPs believed national implementation of pharmacist prescribing would have to be gradual, and probably at first dependent.

PD4: “I think the support would be greater if at first it were dependent prescribing.”

GP7: “For us GPs, it will be difficult to let go of control.”

The pharmacists saw challenges related to training pharmacists and increasing the number of pharmacists who could prescribe. They stressed the need for a national program that would support the implementation and provide technical and administrative support.

They also underlined the challenge of getting GPs on board.

PD2: “Where the medication review has already been well implemented, and they are collaborating well with the GPs, they’d be open to it.”

GP1: “Maybe it would help if the GPs who didn’t participate in the study got some … positive introduction … and that this can eventually become part of every healthcare centre.”

Both pharmacist prescribers and patients also saw the possibility of collaboration with nurses who are part of the primary healthcare teams in Slovenia, doing preventative check-ups and monitoring well-controlled chronic disease patients. During such a check-up, a chronic patient would, in their vision, get a consultation with the pharmacist prescriber and have therapy optimized depending on laboratory tests and other results. Collaboration with nurses would also help with referral and monitoring patients.

PT4: “Once yearly, I have a preventative check-up … and when they have [blood] results, they could also give it to the pharmacist prescriber to see what can be done.”

Both GPs and pharmacist prescribers expected the protocols for individual diseases to be refined further with practice. GPs, in particular, noted that there would have to be gradual progress in competencies for different diseases with exact protocols.

GP6: “Maybe we should start with some areas that are easier to manage … You have to get used to it, and when it starts to run smoothly, you see how good it is.”

One GP, however, was worried that it would be difficult to manage just a single illness or permit prescription of a single medication class in a multimorbid patient with many illnesses and medications.

GP3: “The patient is a whole, I’m not sure if we can divide this so strictly.”

According to the stakeholders, every implementation would ultimately require building collaboration between individual GPs and pharmacist prescribers.

GP5: “I’m not sure if something general will go through. You must [manage] collaboration between a specific GP and a pharmacist prescriber.”

## 4 Discussion

Based on the national clinical pilot trial, this study represents the first qualitative investigation of pharmacist prescribing conducted outside Anglo-Saxon countries based on experiences from a real national pilot. Previous studies in similar settings did not involve pharmacists as prescribers, but rather captured only the opinions of participants who had no direct experience with pharmacist prescribers in primary care (Rose et al., 2025). A mixed-methods study from Austria included community and hospital pharmacists, demonstrating that community pharmacists frequently used clinical judgement in urgent situations. More than 88% of included pharmacists supported an expanded scope of practice, particularly in continuing contraceptive prescriptions, managing chronic diseases, and treating infections using point-of-care testing. Hospital pharmacists reported limited implementation of prescribing frameworks, hindered by institutional inertia, staff shortages, and restricted access to patient data. The authors concluded that additional training and policy support were needed (Rose et al., 2025). Similarly, in France, community pharmacists are authorised to dispense certain antibiotics without a prescription after completing a mandatory 5-h training programme (French Directorate for Legal and Administrative Information, 2025). Despite these positive examples, there remains a paucity of studies examining clinical outcomes and qualitative data on pharmacists prescribing outside the Anglo-Saxon countries.

This study has important practical implications. All stakeholders—patients, pharmacist prescribers, and GPs—expressed positive experiences. Pharmacist prescribers in this pilot programme developed their roles from the medication review service, which has been a standard of care in Slovenia since 2016 and provided a foundation for prescribing activities and long-term monitoring (Stuhec, 2025a; Stuhec et al., 2021). GPs and patients reported trust in the pharmacist prescribers’ competence and prescribing decisions. Comparable findings have been reported in the UK, where most GPs agree that pharmacist prescribers possess the skills and knowledge required for independent prescribing (Hasan Ibrahim et al., 2022; Stewart et al., 2009; Hindi et al., 2019). Patients’ perceptions were also mainly positive in the UK in a study focused on pharmacists and nurses independent prescribers, where researchers reported some barriers, including competencies, organisational factors and support from colleagues (Hindi et al., 2019). Both independent prescribers and GPs (n = 25) strongly agreed that independent prescribing improved patient care quality (Hindi et al., 2019). Stakeholders in this study confirmed that a pilot trial was essential to generate real-world data before wider implementation and attributed their positive perceptions to long-standing collaboration through medication review services, which have already been evaluated qualitatively, quantitatively, and economically in Slovenia (Stuhec, 2025a; Nabergoj Makovec et al., 2023). Based on these findings, researchers in our study incorporated additional patient monitoring and prescribing by clinical pharmacists. This information is particularly valuable for countries seeking to develop pharmacist prescriber roles.

A second key finding relates to clinical outcomes, including sustained patient monitoring. All interviewed participants highlighted improved treatment outcomes and enhanced access to healthcare services. This was shown quantitatively in our previous clinical study involving 191 patients (Stuhec et al., 2025). In the present qualitative study, both patients and GPs highlighted the value of more comprehensive monitoring beyond the standard medication review, noting that this approach was particularly beneficial. GPs and patients strongly endorsed and highly valued the concept of long-term follow-up. This qualitative evidence suggests that pharmacists, working collaboratively with GPs, can effectively monitor patients and optimise therapy (Stuhec et al., 2025). These results align with the Committee of Ministers’ Resolution CM/Res(2020)3 on the implementation of pharmaceutical care to benefit patients and health systems (Committee of Ministers Resolution CM/Res, 2020). Therefore, medication review and longitudinal follow-up until the next consultation are crucial service enhancements, particularly in Slovenia, where clinical pharmacists currently perform reviews without ongoing monitoring (Stuhec, 2025a; Stuhec et al., 2021). Pharmacist prescribers and GPs highlighted the value of including clinical data, laboratory tests, and patient outcomes in future monitoring processes. Similar findings have been reported in the UK, where a systematic review of non-medical prescribing concluded that prescribing is more readily integrated into practice when it forms part of the overall patient care pathway. Conversely, when prescribing involves the creation of entirely new professional roles, adoption tends to be slower and requires more time to become widespread (Graham-Clarke et al., 2019).

Motivation, trust, and interprofessional relationships were also key findings, aligning with the CFIR domains of “Characteristics of Individuals” and the “Inner Setting”. GPs and patients expressed high levels of trust in pharmacist prescribers, consistent with findings from the UK (Stewart et al., 2009; Hindi et al., 2019). Notably, our study’s GPs and pharmacist prescribers emphasised the key role of existing collaborative relationships (the pharmacists had already been working with primary care teams in the medication review form before the study).

GPs valued improved access to care, noting that consultations with other specialists often involve long waiting times. Pharmacist prescribers could help fill this service gap. GPs also reported good communication with pharmacist prescribers—an area that can be challenging with other specialists, such as psychiatrists. For instance, only 22% of German GPs report satisfactory communication with psychiatrists, which they consider a barrier to effective depression management (Lech et al., 2022). Both pharmacists and GPs identified restricted access to patient data as a persistent obstacle. The Ministry of Health is addressing this issue, and starting in 2025, all clinical datasets will be available to clinical pharmacists. They will also be required to upload medication reviews to the central electronic health record, making them accessible to patients, physicians, and pharmacists.

The pilot utilised a CPA model, enabling pharmacists to initiate, discontinue, or switch medications for 10 therapeutic groups and prescribe all medications within the GP’s scope (Stuhec et al., 2025). This model is comparable to New Zealand and the United States (Raghunandan et al., 2021; Pharmacycouncil.org, 2025). GPs and pharmacist prescribers considered this model appropriate and recommended that prescribing privileges should not be overly restrictive.

Legislation and regulatory frameworks emerged as major determinants of implementation. Both GPs and pharmacist prescribers acknowledged that clinical pharmacists in Slovenia do not yet have prescribing rights and recognised this as a critical step before systemic implementation. GPs emphasised that the responsibilities of pharmacist prescribers should be more clearly defined to facilitate successful systemic implementation. Patients also indicated that the role of pharmacist prescribers should be clearly communicated to them to enhance understanding and engagement with the service. The UK followed a similar trajectory, with prescribing rights emerging from early pilot programmes (Stewart et al., 2009). The next step could involve the Ministry of Health proposing legislative amendments to grant provisional (dependent) prescribing rights to clinical pharmacists, clearly defining their professional responsibilities. Stakeholders also stressed the need to train a sufficient workforce to meet service demand, a challenge mentioned in implementation research from the UK (Edwards et al., 2022). A coordinated, staged approach to workforce development would help establish pharmacist prescribing as a sustainable solution to healthcare workforce pressures in Slovenia and internationally.

Stakeholders supported national implementation but identified education and training as key barriers. Similar concerns were reported in UK implementation studies (Hasan Ibrahim et al., 2022). The Slovenian system should therefore define competencies for pharmacist prescribers, building on existing competencies for medication review developed by the Slovenian Chamber of Pharmacy (Stuhec, 2025a). GPs identified this as a barrier, emphasising that pharmacist prescribers must demonstrate appropriate clinical competencies. Similar UK studies have highlighted this requirement, including those involving supplementary (dependent) pharmacist prescribers (Stewart et al., 2009). In the UK, standards for education and training have been established, and by 2026, all pharmacy graduates will qualify with independent prescribing competencies (General Pharmaceutical Council, 2025). Slovenia should adopt a similar approach, potentially drawing on New Zealand’s model (dependent prescriber), where pharmacists must complete postgraduate qualifications and maintain clinical competencies (Raghunandan et al., 2021; General Pharmaceutical Council, 2025). Reimbursement was also identified as a barrier, similar to the UK (Stewart et al., 2009). Pharmacist prescribers and GPs proposed that the Health Insurance Institute of Slovenia negotiate a payment model for pharmacist prescribing, potentially linked to the existing medication review reimbursement (approximately €50 per review) (Stuhec, 2025a).

### 4.1 Strengths and limitations

This study has several important practical implications, particularly for the development of pharmacist prescribers and the integration of long-term patient monitoring in Slovenia. These findings suggest that existing health programmes, such as the medication review service, could be expanded to incorporate longitudinal follow-up, thereby enhancing continuity of care. The most significant implication is the strong stakeholder support for pharmacist prescriber development, which provides a robust foundation for the Ministry of Health of the Republic of Slovenia to implement this service nationally. One limitation of the study is that some co-authors participated in the pilot trial; however, potential bias was minimized because the researchers who conducted the semi-structured interviews and qualitative analysis were not involved in the pilot trial (Dunja Mahoric and Eva Gorup).

Nevertheless, some limitations should be acknowledged. Participants were recruited from pilot trial sites, which may have introduced selection bias; however, this approach was chosen to ensure participants had direct experience with pharmacist prescribing. In addition, the use of semi-structured interviews limited the sample size but enabled rich, in-depth exploration of stakeholder perspectives, which may not have been captured through a cross-sectional survey with a standardised questionnaire. Finally, the study focused exclusively on pharmacist prescribers in primary care, excluding those working in hospitals or community pharmacies. This reflects the pilot project’s primary care focus, where medication review services have been established and reimbursed since 2016.

### 4.2 Conclusion

This is the first qualitative study of pharmacist prescribing based on experiences from national pilot trial conduced outside Anglo-Saxon countries. The results indicate positive experiences and strong support from GPs, pharmacist prescribers, and patients for the introduction of pharmacist prescribing in Slovenia. Addressing barriers—including education, legislation restrictions, and limited electronic health record options will be essential for successful national implementation.

## Data Availability

The original contributions presented in the study are included in the article/Supplementary Material, further inquiries can be directed to the corresponding author.
