# Supplementary material for: Pharmacist prescriber implementation in the experiences of general practitioners, pharmacist prescribers and patients: qualitative study based on pilot trial in Slovenia
Source: Front Pharmacol. 2025 Nov 12;16:1712595. doi: 10.3389/fphar.2025.1712595 (PMC12646872; doi:10.3389/fphar.2025.1712595)
Supplement: Supplementary file 1 [file Table1.docx]

| Interview Questions – Pharmacist prescribers | |
| --- | --- |
| **Domain** | **Question** |
| **Intervention characteristics** | Which international models of pharmacist prescribing are you familiar with? How would you adapt them to your current system and the pilot? |
|  | Which professional guidelines, literature, or training do you rely on in prescribing decisions in the pilot project? |
|  | Would you highlight any barriers or obstacles you most often encounter? |
| **Outer setting** | What do you think about the current legislation – does it fit autonomous or dependent prescribing by pharmacists? Where do you see the most significant advantages and gaps? |
|  | How would patients respond to such an expansion of your responsibilities? Would their motivation for consultation increase or decrease? How did patients and doctors accept this collaboration in the pilot project? |
| **Inner setting** | What kind of support do you feel from management or the medical team? Do you think the culture in your environment is favourable for expanding pharmacist competences from the pilot to a national level? |
|  | How did you find prescribing in the pilot project (safe and effective enough)? Which resources (time, staff, monitoring tools) would you still need? |
| **Characteristics of individuals** | How do you assess your own competence for prescribing? Where do you feel the most significant gaps or need for further training? Is this model suitable, or would an independent prescribing model (like in the UK) be better? Can you tell us more about the current collaborative practice agreement (CPA) – is it valuable and good enough? |
|  | How is your communication with general practitioners? Do you feel the relationship (in the pilot) is sufficiently collaborative to see you as an equal partner in prescribing? |
| **Process** | What would be crucial for introducing the service into the system – regular meetings, pilot clinics, or working groups? How would you like to be involved in planning? |
|  | How would you monitor and evaluate treatment success (as in the pilot)? Would you use indicators such as the number of consultations, changes in adherence, and satisfaction? Would you monitor clinical outcomes, and is this meaningful? Why do you consider this an upgrade of the current collaboration model (medication review in primary care settings)? |

| Interview Questions – Patients | |
| --- | --- |
| **Domain** | **Question** |
| **Intervention characteristics** | Before participating in the pilot, were you aware that a pharmacist could also be involved in decisions about the medicines you receive? Do you see this as an advantage or a disadvantage? |
|  | Do you think it makes a difference whether a pharmacist or a general practitioner (GP) prescribes your medicine, if you have enough information? Can you tell us more about this? |
| **Outer setting** | How did you respond when a pharmacist prescribed you medicines (was it, in your opinion, of good quality, and did it contribute to faster care)? Can you describe your case and experience? |
|  | In some countries (e.g., New Zealand), this collaboration already exists. Have you ever heard of such examples abroad? How much trust would you have in their professionalism and accessibility? |
| **Inner setting** | Are you aware of how communication between your GP and pharmacist works? Would it reassure you that they work together as a team in your therapy? |
|  | Have you ever felt that there was insufficient time in the GP’s office to explain medicines? Would you also prefer to be able to turn to a pharmacist to discuss a medicine and possibly receive a prescription? Did the pharmacist explain this to you in the pilot, and was it helpful (please give an example)? |
| **Characteristics of individuals** | How important is it for you personally to be involved in decisions about your medicines? Do you feel more included if the pharmacist provides more information and guidance? |
|  | Did it matter to you whether a GP or a pharmacist prescribed the medicine? |
| **Process** | In your opinion, is the current collaboration suitable for integration into the Slovenian healthcare system? How do you imagine the procedure: would you need a referral from a doctor, or could you visit a pharmacist with prescribing authority directly? |
|  | How do you think we (researchers) could best measure whether you were satisfied with this model of care? For example, would questionnaires, interviews, or ongoing follow-up by the pharmacist be appropriate? Did your health improve after collaborating with the pharmacist prescriber (please give an example)? Do you trust the pharmacist as a prescriber? |

| Interview Questions – General practitioners | |
| --- | --- |
| **Domain** | **Question** |
| **Intervention characteristics** | How do you assess the added value of pharmacists being able to prescribe medicines? What benefits do you see for your practice and patients? Can you tell us more about upgrading from the model where pharmacists only wrote a report to the one where they can also prescribe? |
|  | Which aspects of such a model seem most challenging to you? Would you highlight any barriers or obstacles you most often encounter? |
| **Outer setting** | What are, in your opinion, the legal and financial conditions for the successful implementation of pharmacist prescribing? What should be regulated or changed? |
|  | How do you think most patients would accept a pharmacist as a prescriber? Have patients expressed any concerns? Would this improve access to therapy and quality of care? Do you support the idea, as in New Zealand, that prescriptions do not need to be confirmed by a doctor (so the patient would receive the medicine immediately after the pharmacist’s consultation)? |
| **Inner setting** | How is collaboration between GPs and pharmacists organised in your health centre within the project? Is there a protocol that enables task sharing in prescribing? How does patient referral work, and how do patients respond? |
|  | Which resources (time, IT support, protocols) should be available for this prescribing model to run smoothly? How do you perceive current collaboration within the pilot project? |
| **Characteristics of individuals** | How much do you trust the competencies of pharmacists compared with those of GPs? Which cases would you hand over to pharmacists without hesitation (e.g., the same groups of medicines as in the pilot project)? Can you tell us more about the collaborative practice agreement (CPA) – is it broad enough or too narrow in terms of drug groups, and does it give the GP enough freedom to decide which cases to authorize the pharmacist for? |
|  | What is your opinion on sharing responsibility for the safety and effectiveness of therapy if a pharmacist is involved in prescribing? Do you think pharmacists effectively monitor pharmacotherapy and optimize treatment? |
| **Process** | Do you think it would be reasonable to introduce dependent prescribing gradually, as in the pilot project (with mutual agreements, initially only for certain medicines or target groups of patients), or do you think such an agreement is unnecessary (as in the UK, where pharmacists can prescribe without prior agreement)? What do you think about pharmacists initiating therapy after diagnosis is established? What about referral of more complex cases? |
|  | How would you measure the success of such a model? Would you be interested in indicators such as improved clinical outcomes (e.g., reduced HbA1c, remission, improved quality of life), reduced polypharmacy, and patient satisfaction? |
