# Supplementary material for: Pharmacist prescriber implementation in the experiences of general practitioners, pharmacist prescribers and patients: qualitative study based on pilot trial in Slovenia
Source: Front Pharmacol. 2025 Nov 12;16:1712595. doi: 10.3389/fphar.2025.1712595 (PMC12646872; doi:10.3389/fphar.2025.1712595)
Supplement: Supplementary file 2 [file Table2.docx]

CODEBOOK

Pharmacist prescriber

| CIFR domain | Subcodes |
| --- | --- |
| Intervention | Innovation source  Evidence-based (Use of guidelines and protocols)  Adaptability  Complexity  Relative advantage |
| Outer Setting | Legislation and access  Patients' needs, acceptance and expectations |
| Inner Setting | Collaboration with physicians  Communication with the patients  Sharing expertise  Coordination and IT barriers  Available resources (need for technical and administrative support)  Effectiveness |
| Characteristics of Individuals | Expertise and training needs  Motivation (attitude towards prescribing/responsibility) |
| Process | Monitoring and outcomes  National implementation |

General practitioners

| CIFR domain | Subcodes |
| --- | --- |
| Intervention | Acceptability  Complexity |
| Outer Setting | Patient needs/satisfaction  Legislation/Access  Professional responsibility  Quality of Care |
| Inner Setting | Collaboration between general practitioner and pharmacist  Communication with the pharmacist  IT support  Process structure Patient engagement |
| Characteristics of Individuals | Capability/trusting the pharmacist  Knowledge and expertise  Interprofessional relationship  Effectiveness |
| Process | Monitoring and evaluation  Sustainability  National implementation |

Patients

| CIFR domain | Subcodes |
| --- | --- |
| Intervention | Acceptability  Complexity  Relative advantage |
| Outer Setting | Patient needs  Legislation/access |
| Inner Setting | Collaboration between general practitioner and pharmacist  Communication  Process structure |
| Characteristics of Individuals | Trust in the pharmacist  Knowledge and expertise |
| Process | Patient engagement  Sustainability  National implementation |
