# Supplementary material for: Pharmacist prescriber implementation in the experiences of general practitioners, pharmacist prescribers and patients: qualitative study based on pilot trial in Slovenia
Source: Front Pharmacol. 2025 Nov 12;16:1712595. doi: 10.3389/fphar.2025.1712595 (PMC12646872; doi:10.3389/fphar.2025.1712595)
Supplement: Supplementary file 3 [file Table3.docx]

**Supplementary File: Consolidated Criteria for Reporting Qualitative Studies (COREQ): 32-item checklist^a^**

| **No** | **Item** | **Guide questions/Description** | **Response/Page number** |
| --- | --- | --- | --- |
| **Domain 1: Research team and reflexivity** | | | |
| Personal Characteristics | | | |
| 1. | Interviewer/facilitator | Which author/s conducted the interview or focus group? | EG and DM conducted the interviews. |
| 2. | Credentials | What were the researcher's credentials? *E.g., PhD, MD* | Master of Pharmacy – Clinical Pharmacy resident and Doctor of Medicine, Family Medicine Specialist with a PhD. |
| 3. | Occupation | What was their occupation at the time of the study? | Master of Pharmacy working in a public pharmacy, and a Family Medicine physician in general practice and a researcher. |
| 4. | Gender | Was the researcher male or female? | Both researchers were female. |
| 5. | Experience and training | What experience or training did the researcher have? | DM had 23 years of experience as a pharmacist in a public pharmacy and was undergoing specialization in clinical pharmacy. EG – had 20 years of experience as a general practitioner in a public primary care settngs and as assistant professorr from familly medicine at the Facculty of Medicine, University of Ljubljana |
| Relationship with participants | | | |
| 6. | Relationship established | Was a relationship established prior to study commencement? | There was no prior relationship. |
| 7. | Participant knowledge of the interviewer | What did the participants know about the researcher? e.g., personal goals, reasons for doing the research | Some participants knew the researchers’ educational background and their workplace. |
| 8. | Interviewer characteristics | What characteristics were reported about the interviewer/facilitator? e.g., Bias, assumptions, reasons, and interests in the research topic | No characteristics were reported about EG and DM. |
| Domain 2: Study Design | | | |
| Theoretical framework | | | |
| 9. | Methodological orientation and Theory | What methodological orientation was stated to underpin the study? e.g., grounded theory, discourse analysis, ethnography, phenomenology, content analysis | The study employed qualitative content analysis (thematic/framework approach), using the updated Consolidated Framework for Implementation Research (CFIR) domains and constructs. |
| Participant selection | | | |
| 10. | Sampling | How were participants selected? e.g., purposive, convenience, consecutive, snowball | Participants were selected using a purposive sampling method. |
| 11. | Method of approach | How were participants approached? e.g., face-to-face, telephone, mail, email | Participants were invited by telephone, email, and in person. |
| 12. | Sample size | How many participants were in the study? | There were 17 participants in the study. |
| 13. | Non-participation | How many people refused to participate or dropped out? Reasons? | All invited participants agreed to take part. |
| Setting | | | |
| 14. | Setting of data collection | Where was the data collected? e*.g., home, clinic, workplace* | Data was collected at home in a quiet setting via the Zoom platform. |
| 15. | Presence of non-participants | Was anyone else present besides the participants and researchers? | No, there were no other people present. |
| 16. | Description of sample | What are the important characteristics of the sample? *e.g., demographic data, date* | The sample included general practitioners, pharmacist prescribers, and patients with chronic diseases. |
| Data collection | | | |
| 17. | Interview guide | Were questions, prompts, guides provided by the authors? Was it pilot tested? | The questions were not shared with participants beforehand and were pilot tested. |
| 18. | Repeat interviews | Were repeat interviews carried out? If yes, how many? | No, there were no repeat interviews. |
| 19. | Audio/visual recording | Did the research use audio or visual recording to collect the data? | Audio recordings were used for data collection. |
| 20. | Field notes | Were field notes made during and/or after the interview or focus group? | EG and DM took field notes during the interview. |
| 21. | Duration | What was the duration of the interviews or focus group? | The interviews lasted between 20 and 60 minutes. |
| 22. | Data saturation | Was data saturation discussed? | Data saturation was discussed; the number of interviews per group was adjusted until similar responses began to recur. |
| 23. | Transcripts returned | Were transcripts returned to participants for comment and/or correction? | No, the transcripts were not returned to participants. |
| Domain 3: Analysis and findings | | | |
| Data analysis | | | |
| 24. | Number of data coders | How many data coders coded the data? | EG and DM coded the data. |
| 25. | Description of the coding tree | Did authors provide a description of the coding tree? | Coding was based on CFIR domains and constructs. |
| 26. | Derivation of themes | Were themes identified in advance or derived from the data? | Themes were not predetermined, but rather emerged per the CFIR framework. |
| 27. | Software | What software, if applicable, was used to manage the data? | MAXQDA software was used to support data analysis. |
| 28. | Participant checking | Did participants provide feedback on the findings? | No, participants did not provide feedback on the findings. |
| Reporting | | | |
| 29. | Quotations presented | Were participant quotations presented to illustrate the themes / findings? Was each quotation identified? e*.g., participant number* | Participant quotes were included. |
| 30. | Data and findings consistent | Was there consistency between the data presented and the findings? | The data aligned with the findings. |
| 31. | Clarity of major themes | Were major themes clearly presented in the findings? | The major themes were presented clearly. |
| 32. | Clarity of minor themes | Is there a description of diverse cases or discussion of minor themes? | Various cases and minor themes were reported. |

^a^Tong A, Sainsbury P, Craig J. Consolidated criteria for reporting qualitative research (COREQ): a 32-item checklist for interviews and focus groups. International journal for quality in health care. 2007 Dec 1;19(6):349-57.
