# Supplementary material for: Pharmacist prescriber implementation in the experiences of general practitioners, pharmacist prescribers and patients: qualitative study based on pilot trial in Slovenia
Source: Front Pharmacol. 2025 Nov 12;16:1712595. doi: 10.3389/fphar.2025.1712595 (PMC12646872; doi:10.3389/fphar.2025.1712595)
Supplement: Supplementary file 4 [file Table4.docx]

| **Phase** | **Activity / Step** | **Details** |
| --- | --- | --- |
| **I. DESIGN AND PREPARATION** | **1. Formation of the working group and development of the tool** | A dedicated working group developed and piloted the interview guide. The updated Consolidated Framework for Implementation Research (CFIR) was applied to explore barriers and facilitators. |
| **II. DATA COLLECTION** | **2. Sampling and recruitment** | Purposive sampling was used. A total of 17 participants were invited (4 pharmacist prescribers, 8 general practitioners, and 5 patients). Recruitment took place between May and August 2025 until data saturation was achieved. |
|  | **3. Conducting interviews** | Semi-structured interviews were conducted by two researchers who had not participated in the pilot project. Interviews were held via telephone or the Zoom platform. All interviews were audio-recorded and lasted between 20 and 60 minutes. |
| **III. DATA PROCESSING** | **4. Transcription and identification** | Audio recordings were imported into MAXQDA® software and automatically transcribed using MAXQDA® software. Participants were anonymized using numerical identifiers. |
| **IV. DATA ANALYSIS** | **5. Coding (thematic analysis)** | Thematic analysis was applied. Two researchers (Eva Gorup and Dunja Mahorič) independently coded the transcripts. Coding was guided by the five CFIR domains and their associated constructs. |
|  | **6. Discrepancy resolution and consensus** | In case of disagreements, researcher M.S. (Matej Štuhec) served as the final adjudicator. Discrepancies were resolved through negotiated consensus. The working group met to ensure consistency between the data and the emerging findings. |
|  | **7. Deriving themes and reporting** | Themes were derived inductively from the data in alignment with the CFIR framework. Double coding was used to ensure trustworthiness. The final coding framework was confirmed through team consensus. |

Simplified Flow Diagram of Data Collection and Analysis
